# Manifestaciones temporomandibulares en pacientes con síndrome de Ehlers-Danlos: una revisión sistemática

**DOI:** 10.21142/2523-2754-1103-2023-164

**Published:** 2023-09-26

**Authors:** Javiera Cancino, Felipe Soto, Sebastián Martinez, Sergio Gutiérrez

**Affiliations:** 1 Facultad de Odontología, Universidad Finis Terrae. Santiago, Chile. jcancinog@uft.edu, sgutierrezb@uft.edu Universidad Finis Terrae Facultad de Odontología Universidad Finis Terrae Santiago Chile; 2 Escuela de Odontología, Universidad Mayor. Santiago, Chile. felipei.sotodonoso@gmail.com Universidad Mayor Escuela de Odontología Universidad Mayor Santiago Chile felipei.sotodonoso@gmail.com; 3 Facultad de Odontología, Universidad del Desarrollo. Santiago, Chile. sebamartinezven@outlook.com Universidad del Desarrollo Facultad de Odontología, Universidad del Desarrollo Santiago Chile sebamartinezven@outlook.com

**Keywords:** Ehlers-Danlos, síndrome, temporomandibular, articulación, Ehelers-Danlos, syndrome, temporomandibular, joint

## Abstract

**Introducción:**

El Síndrome de Ehlers-Danlos (SED) consiste en un grupo de enfermedades que implican un desorden de los tejidos conectivos de los individuos como producto de una alteración en la síntesis de colágeno. Las estructuras fibrocartilaginosas, los ligamentos de soporte, el disco y el tejido retrodiscal de la articulación temporomandibular (ATM) están compuestos por colágeno.

**Objetivo:**

Identificar las manifestaciones temporomandibulares en pacientes con diferentes subtipos de SED a través de una revisión sistemática. Materiales y métodos: Se realizó una revisión sistemática de la literatura incluyendo las bases de datos de PubMed, Scopus y Web of Science. Se incluyeron estudios observacionales y series de casos en idioma inglés o español publicados hasta enero de 2023.

**Resultados:**

Se seleccionó 12 artículos cumplieron con los criterios de inclusión. La prevalencia de desórdenes temporomandibulares varió entre el 26,6% y el 100%, con el subtipo SEDh como el más reportado. El desorden temporomandibular más identificado fue el desplazamiento del disco con o sin reducción e hipermovilidad articular.

**Conclusión:**

Las patologías temporomandibulares son habituales en pacientes con SED, especialmente en aquellos con el subtipo SEDh.

## Abstract (en)

**Introduction:**

Ehlers-Danlos Syndrome (EDS) consists of a group of diseases that imply a disorder among individuals connective tissue due to an altered collagen production. Fibrocartilaginous structures, support ligaments, disc and retrodiscal from the temporomandibular joint are composed primarily of collagen.

**Objective:**

Identify TMJ manifestations in patients with different types of EDS. Materials and Methods: a systematic review of the literature was performed on PubMed, Scopus and Web of Science databases. Observational studies and case series reports published either in spanish or panish were included up until January 2023.

**Results:**

12 articles met the inclusion criterial. The prevalence of temporomandibular disorders varies from 26.6% to 100%, being EDSh the most reported subtype, whereas disc displacement with or without reduction and joint hypermobility were the most reported joint disorders.

**Conclusion:**

Temporomandibular pathologies are common in patients with EDS, especially in those with the EDSh subtype.

## INTRODUCCIÓN

El Síndrome de Ehlers-Danlos (SED) consiste en un grupo de enfermedades que implican un desorden de los tejidos conectivos de los individuos como producto de una alteración en la síntesis de colágeno ^1^. Se estima una prevalencia de 10 casos entre 5000 pacientes, y las afectadas son principalmente mujeres. Existen 13 subtipos de SED, de los cuales el SEDh es el más frecuente (80-90% de los pacientes) ^2^. Su fisiopatología involucra una serie de mutaciones heredadas en la síntesis y el procesamiento de colágeno, el cual es una parte integral de la función de cada sistema corporal, desde la piel hasta la integridad vascular ^3^. Clínicamente, se caracteriza por presentar la tríada de hipermovilidad articular, hiperextensibilidad cutánea y friabilidad del tejido conectivo ^4^. 

El consorcio internacional de SED, formado en 2012, ideó en 2017 una clasificación del SED que estableció 13 subtipos, de acuerdo con sus manifestaciones clínicas y criterios clínicos mayores y menores, los cuales proveen una alta especificidad diagnóstica ^5^ (Tabla 1).

**Tabla 1:** Clasificación Internacional de síndromes Ehlers-Danlos, 2017

Subtipo	Principales afecciones genéticas	Características clínicas mayores
SED clásico (SEDc)	COL5A1	a. Hiperextensibilidad cutánea y cicatrización atróficas
		b. Hipermovilidad articular generalizada
SED tipo-clásico (SEDtc)	TNXB	a. Hiperextensibilidad cutánea con textura aterciopelada sin cicatrización atrófica
		b. Hipermovilidad articular generalizada con/sin dislocaciones recurrentes (principalmente hombros y tobillos)
		c. Equimosis espontáneas
SED cardiovascular (SEDcv)	COL1A2	a. Cardio-vasculopatías progresivas severas (aórtica y mitral)
		b. Hiperextensibilidad cutánea, cicatrización atrófica, piel delgada y equimosis de fácil desarrollo
		c. Hipermovilidad articular (generalizada o exclusivamente en pequeñas articulaciones)
SED vascular (SEDV)	COL3A1	a. Historia familiar de vEDS con variante causante COL3A1 documentada
		b. Ruptura arterial en temprana edad
		c. Perforación espontánea de colon sigmoides sin diagnóstico de enfermedad diverticular u otra patología intestinal
		d. Ruptura uterina durante el tercer trimestre sin cesárea previa y/o periparto con desgarros severos del perineo
		e. Formación de fístula del seno carotídeo-cavernoso en ausencia de trauma
SED hipermóvil (SEDh)	Desconocido	a. Hipermovilidad articular generalizada predisponente de inestabilidad y enfermedad articular degenerativa precoz
		b. Leve hiperextensibilidad cutánea
		c. Equimosis de fácil producción
		d. Disautonomía
		e. Fatiga y dolor crónico
		f. Problemas funcionales gastrointestinales
SED artrocalasia (SEDa)	COL1A1, COL1A2	a. Dislocación de cadera congénita bilateral
		b. Hipermovilidad articular generalizada severa con múltiples dislocaciones/subluxaciones.
		c. Hiperextensibilidad cutánea
SED dermatosparaxis (SEDd)	ADAMTS2	a. Fragilidad cutánea extrema con desgarros de piel congénitos o postnatales
		b. Rasgos craneofaciales característicos, que son evidentes al nacer, en infancia temprana o tardía
		c. Piel redundante, casi laxa, con pliegues excesivos en las muñecas y tobillos
		d. Arrugas palmares aumentadas
		e. Equimosis severas con riesgo de hematomas y hemorragia subcutánea
		f. Hernia umbilical
		g. Crecimiento postnatal retardado
		h. Extremidades, manos y pies cortos
		i. Complicaciones perinatales debido a fragilidad de tejido conectivo
SED cifoescoliótico (SEDcf)	PLOD1, FKBP14	a. Hipotonía muscular congénita
		b. Cifoescoliosis congénita o de inicio temprano (progresiva o no progresiva)
		c. Hipermovilidad articular con dislocaciones/subluxaciones (hombros, caderas y rodillas particularmente)
Sindrome córnea frágil	ZNF469, PRDM5	a. Córnea delgada con o sin ruptura (grosor de central de córnea < 400 mm)
		b. Queratocono progresivo de temprana aparición
		c. Queratoglobo progresivo de temprana aparición
		d. Esclera azul
EDS espondilodisplásico (SEDep)	B4GALT7, B3GALT6, SLC39A13	a. Estatura pequeña (progresiva en la infancia)
		b. Hipotonía muscular (desde severo congénito hasta inicio tardío moderado)
		c. Arqueamiento de extremidades
SED musculocontractural (SEDmc)	CHST14, DSE	a. Múltiples contracturas congénitas, características contracturas de aducción-flexión, pie de zambo (talipes equinovarus)
		b. Rasgos craneofaciales característicos, evidenciables al nacer o temprana infancia.
		c. Rasgos cutáneos característicos incluyendo hiperextensibilidad de la piel, equimosis de fácil desarrollo, fragilidad cutánea con cicatrices atróficas, arrugas palmares aumentadas
SED miopático (SEDm)	COL12A1	a. Hipotonía muscular congénita y/o atrofia muscular que aumenta con la edad.
		b. Contracturas de articulaciones proximales (rodillas, caderas y codos)
		c. Hipermovilidad de articulaciones distales
SED periodontal (SEDp)	C1S, C1R	a. Periodontitis severa e intratable de inicio temprano (infancia o adolescencia)
		b. Encía adherida deficiente
		c. Placas pretibiales
		d. Antecedentes familiares de un pariente de primer grado que cumple los criterios clínicos

La articulación temporomandibular (ATM) es un tipo de articulación sinovial, ginglimoartrodial, bicondílea doble, que permite movimientos de traslación y, en menor medida, de rotación ^6^. Histológicamente, los componentes de la ATM contienen fibroblastos y células tipo condroblastos, por lo que la síntesis de colágeno es un factor primordial en la estructura y función de esta articulación. Sin embargo, la característica que la hace única es que las superficies articulares están cubiertas por fibrocartílago en lugar de cartílago hialino, como en otras articulaciones ^7^.

El colágeno y su función se ven alterados en todos los subtipos del SED y pueden causar síntomas en diferentes zonas del complejo orofacial ^8^. Específicamente, en la ATM, las estructuras fibrocartilaginosas, los ligamentos de soporte, el disco y el tejido retrodiscal están compuestos por colágeno ^9^ y una mayor alteración puede presentarse. Por ello, el objetivo de este trabajo fue identificar las manifestaciones temporomandibulares en pacientes con diferentes subtipos de SED a través de una revisión sistemática. 

## MATERIALES Y MÉTODOS

### Registro y protocolo

Una búsqueda sistemática de la literatura fue realizada acorde a las pautas “Preferred Reporting Items for Systematic Review and Meta-Analysis” por sus siglas en inglés, PRISMA ^10^, y registrada en PROSPERO con el código de registro de CRD42022382171. 

### Estrategia de búsqueda y criterios de selección

Una búsqueda general fue realizada en las bases de datos PubMed, Scopus y Web of Science. Las referencias de los estudios incluidos también fueron revisadas para encontrar estudios faltantes. Las palabras de búsqueda fueron *Ehlers-Danlos* y *temporomandibular*.

La búsqueda y la selección de estudios se basaron en la población, el factor pronóstico y el resultado “*outcome*” (PFO) ^11^, donde:

- Participantes: población adulta o niños. 

- Factor pronóstico: la exposición de interés aborda la presencia del síndrome de Ehlers-Danlos evaluada mediante examen genético, físico o cuestionario.

- *Outcome*: manifestaciones temporomandibulares. 

Los diseños de los estudios incluidos fueron caso control, transversal y serie de casos. No se consideró una fecha límite inferior, mientras que el límite superior fue enero de 2023. Los estudios excluidos fueron las revisiones narrativas, los comentarios, los estudios en animales o aquellos cuyos datos no podían ser extraídos de forma fiable.

Así, se concluyó utilizar las siguientes estrategias de búsqueda en las respectivas bases de datos: 

#### PubMed:

("Ehlers-Danlos" OR "EDS " OR "Ehlers Danlos" OR "Ehlers-Danlos Syndrome"[Mesh] OR Joint hypermobility syndrome) AND ("Temporomandibular" OR "Temporomandibular Joint Disfunction" OR "TMJD "OR" Temporomandibular Joint Disorders" [Mesh] OR "TMJ") 

#### Scopus: 

TITLE-ABS-KEY (Ehlers Danlos) OR TITLE-ABS-KEY (eds) OR TITLE-ABS-KEY (Ehlers AND Danlos) AND TITLE-ABS-KEY (temporomandibular) OR TITLE-ABS-KEY (temporomandibular AND joint AND disfunction) OR TITLE-ABS-KEY (tmj) OR TITLE-ABS-KEY (TMJ)

#### Web of Science:

(ALL=(Ehlers Danlos) OR ALL=(EDS) OR ALL=(Ehlers-Danlos)) AND (ALL=(temporomandibular) OR ALL=(temporomandibular joint disorder) AND ALL=(TMJ) AND ALL=(TMJD))

### Evaluación screening y selección de estudios

Todas las referencias fueron organizadas y subidas en el programa Endnote 20 para remover duplicados. Dos revisores independientes (JC y FS) fueron los encargados de evaluar los estudios recopilados mediante título y *abstract*, en función de los criterios de selección. Se incluyó todo artículo completo de los títulos que cumplieran aparentemente con los criterios de inclusión. En caso de existir un desacuerdo, un tercer revisor (SG) lo resolvió mediante discusión. 

### Extracción de datos

La extracción de datos fue ejecutada de forma independiente por dos autores (JC y FS). La información esencial que fue extraída de los estudios incluidos fue la siguiente: nombre del primer autor, año de publicación, país de la población participante, diseño de estudio, número de participantes, sexo y edad de los participantes, subtipo de Ehlers-Danlos, confirmación diagnóstica y trastornos temporomandibulares. Las contradicciones reportadas durante la extracción de datos fueron removidas en un consenso al final de la revisión independiente. La extracción final fue preparada en una tabla de Microsoft Excel para su análisis (Tabla 2).

**Tabla 2:** Características de los estudios incluidos en la revisión sistemática

Autor, país y año	Diseño de estudio	Número de pacientes	Sexo Edad en años, Promedio±DS (Rango)	Tipo EDS /Confirmación diagnóstica	Disfunción temporomandibular		
					Evaluación TMJ	Prevalencia DTM	Signos y síntomas
Bech *et al*. Dinamarca, 2022	Caso-Control	Casos 26	F 22	hEDS 100%	Examen clínico con criterios DC/TMD	ND	Dolor durante apertura, lateralidad y protrusión
		Controles 39	M 4	/Test genético mediante muestra salival			Crépito y clic articular
			34,5 ± 10,1 años				Bloqueo articular
			(R 20; 50)				Mialgia
							Dolor miofascial con dolor referido
			Controles ND*				Artralgia
							Dolor de cabeza atribuido a DTM
							Desplazamiento del disco con reducción
							Desplazamiento del disco con reducción con apertura limitada
							Desplazamiento del disco sin reducción y sin apertura limitada
							Enfermedad articular degenerativa
Song *et al.* Estados Unidos, 2021	Transversal	98	F 96%	hEDS 67%	Registro médico	35,7%	
			M 4 %	cEDS 1%			
			37,5 ± 11,8	vEDS 1%			
			(R 18; 67)	NE 20%			
				/Registro médico			
Glayzer *et al*. Estados Unidos, 2021	Transversal	1146	F 100%	hEDS 91.3%	Auto reporte	56,4%	
			38,2 ± 11,5	cEDS 4.4%			
				vEDS 9%			
				tcEDS 8%			
				kEDS 5%			
				cvEDS 2%			
				aEDS 1%			
				mEDS 1%			
				No está seguro 1,8%			
				/Autorreporte			
Van Camp *et al*. Bélgica, 2020	Serie de caso	21	F 18	ND	Autorreporte	85,7%	
			M 3	/Autorreporte			
Hanisch *et al*. Alemania, 2020	Transversal	79	F 83,5%	ND	Autorreporte	26,6%	
			M 16,5%	/Autorreporte			
			38 (R 16; 81)				
Di Giacomo *et al*. Italia, 2017	Transversal	45	ND	ND/ ND	Exámen clínico/ DC/TMD	84,4%	Desplazamiento del disco sin reducción
							Subluxación
							Artralgia
							Mialgia
Ferré *et al*. Francia, 2012	Caso-control	Casos 17	Casos	vEDS 100% /Confirmación genética	Examen clínico RDC/TMD	82%	Desórdenes musculares
		Controles 46	F 12				Desplazamiento del disco
			M 5				Artralgia, artrosis, artritis
			33 (R 24; 44)				Dolor
		
			Controles				
			F 33				
			M 13				
			36 (R 25; 45)				
Jerjes *et al*. Reino Unido, 2010	Serie de caso	18	F 100%	ND	Clasificación de Wilkes	100%	Estadio II 5 (27,8)
			34 (R 23; 60)	/Derivación por reumatólogo			Estadio III 9 (50,0)
							Estadio IV 3 (16,7)
							Estadio V 1 (5,5)
Castori *et al*. Italia, 2010	Transversal	21	F 85,7%	hEDS 100%	Examen físico	57,1%	
			M 14,3%	/Criterios Villefranche y Brighton			
De Coster *et al*. Bélgica, 2005	Caso-control	Casos 31	Casos	hEDS 16	RDC/TMD	100%	Desórdenes musculares (dolor miofascial)
		Controles 49	F 65%	cEDS 9			Desórdenes del disco unilateral y bilateral con reducción
			M 35%	vEDS 6			Artralgia uni o bilateral
			28±15,3 (R 4; 61)	/Registros médicos			Dolor
							ND
			Controles				
			F 61%				
			M 39%				
			29,2 ± 15,9 (R 5; 62)				
Hagberg *et al*. Suecia, 2004	Caso-control	Casos 114	Casos	No sabe 46%	Auto reporte	44%	Dolor músculos de la masticación
		Controles 114	44 (R 42; 46)	hEDS 32%			Hipermovilidad articular durante la apertura
				pEDS 6%			Bloqueo articular
			Controles	gEDS 6%			Apertura limitada
			44 (R42; 45)	mEDS 4%			Clic articular
				aEDS 4%			Crépito articular
Hagberg *et al*. Suecia, 2004	Caso-control	Casos 144	Casos	No sabe 51%	Autorreporte	45%	Dolor en músculos de la masticación
		Controles 331	F 126	hEDS 31%			Problemas temporomandibulares
			M 18	pEDS 6%			Hipermovilidad articular
				gEDS 5%			
			Controles	aEDS 4%			
			F 226	mEDS 3%			
			M 65				
		/Autorreporte					

ND: No descrito; F: Femenino; M: Masculino; DS: Desviación estándar; R: Rango

### Calidad de los estudios

La calidad de la evidencia fue evaluada de forma independiente por dos autores (JC y SG) usando la herramienta de análisis de sesgos NIH (National Institutes of Health) para estudios de caso-control, transversales y series de caso. Esta herramienta está validada para evaluar la calidad de evidencia en estudios no randomizados. La calidad de los estudios es catalogada, según el puntaje obtenido, en buena, moderada y pobre.

## RESULTADOS

**Figura 1:**
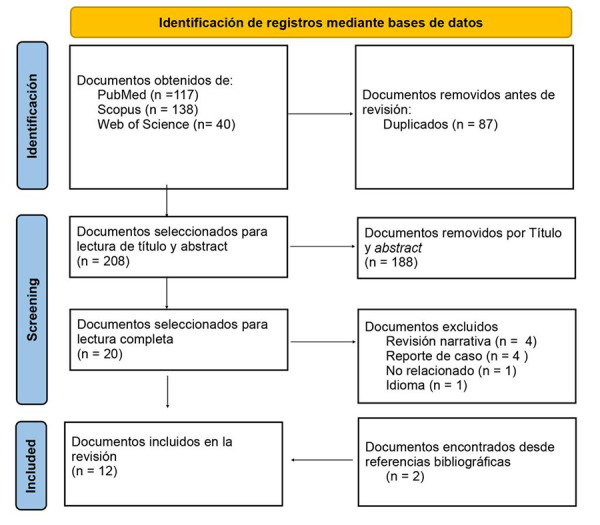
Diagrama de flujo de los artículos seleccionados

La revisión sistemática alcanzó un total de 295 artículos, de los cuales 87 fueron duplicados. Se filtró por medio del título y resumen los 208 documentos restantes, y se eliminó un total de 188 artículos. Una revisión completa de los 20 estudios restantes arrojó que 8 no cumplieron satisfactoriamente los criterios de inclusión. Así, en total, 12 artículos fueron elegibles e incluidos en la revisión sistemática. El diagrama de flujo PRISMA muestra el proceso de revisión y selección (Figura 1).

La calidad de los artículos puede observarse en las tablas 3 a 5. Del total de los artículos seleccionados, 6 fueron catalogados como calidad moderada y 6 como de pobre calidad. Ningún artículo fue evaluado como de buena calidad.

**Tabla 3:** Resultado del análisis de sesgo de los estudios con la escala NIH para estudios caso control

Autor/Pregunta	P1	P2	P3	P4	P5	P6	P7	P8	P9	P10	P11	P12	P13	Calidad
Bech *et al*. Dinamarca, 2022	S	S	NR	S	N	S	S	N	N	S	S	N	N	Moderada
Ferré *et al*. Francia, 2012	S	S	NR	N	N	S	S	N	N	S	S	N	S	Moderada
De Coster *et al*. Bélgica, 2005	S	N	NR	N	N	S	S	N	N	S	N	N	S	Moderada
Hagberg *et al*. Suecia, 2004	S	S	NR	S	N	S	S	N	S	S	N	N	S	Moderada
Hagberg *et al*. Suecia, 2004	S	S	NR	N	N	S	S	N	S	S	N	N	S	Moderada

P1: ¿Fue la pregunta de investigación u objetivos claros y apropiados? P2: ¿Estaba la población de estudio especificada y definida claramente? P3: ¿Fue definida la población blanco y fueron los casos representativos de esta? P4: ¿Incluyó el autor justificación del tamaño de muestra? P5: ¿Fueron los controles seleccionados de la misma o similar población de la que se obtuvo los casos (incluyendo el mismo periodo de tiempo)? P6: ¿Hubo definiciones, criterios de inclusión o exclusión, algoritmos o procesos válidos, fiables y consistentes utilizados para identificar los casos y controles a lo largo del estudio? P7: ¿Fueron los casos claramente definidos y diferenciados de los controles? P8: Si menos del 100% de los casos y controles elegibles fueron seleccionados para el estudio, ¿fueron los casos/controles elegidos aleatoriamente? P9: ¿fueron usados controles concurrentes? P10: ¿Pudieron lo investigadores confirmar que la exposición/riesgo ocurrió antes del desarrollo de la condición o evento que define a los participantes como caso? P11: ¿Fueron las medidas de exposición/riesgo claramente definidas, validas, fiables e implementadas consistentemente? P12: ¿Fueron los investigadores ciegos a la exposición/riesgo de los casos/controles que participaron en el estudio? P13: ¿Se midieron las potenciales variables confundidoras y ajustadas en el análisis estadístico? Si se usó pareamiento, ¿los investigadores reportaron esto durante el análisis del estudio? S: Sí; N: No: NA: No aplica; NR: No reportado. Pobre: 0-4 criterios. Moderada: 5-9 criterios. Buena: 10-13 criterios.

**Tabla 4:** Valoración del riesgo de sesgo según la escala NIH para estudios transversales

Autor/Pregunta	P1	P2	P3	P4	P5	P6	P7	P8	P9	P10	P11	P12	P13	P14	Calidad
Song *et al*. Estados Unidos, 2021	S	N	NC	NC	N	N	N	NA	NA	NA	S	NA	NA	N	Pobre
Glayzer *et al*. Estados Unidos, 2021	S	N	N	N	N	N	N	NA	NA	NA	S	NA	NA	N	Pobre
Hanisch *et al*. Alemania, 2020	S	S	S	S	N	N	N	NA	NA	NA	N	NA	NA	N	Pobre
Di Giacomo *et al*. Italia, 2017	S	S	NC	S	N	N	N	NA	NA	NA	S	NA	NA	N	Pobre
Castori *et al*. Italia, 2010	S	S	NA	S	N	N	N	NA	NA	NA	N	NA	NA	N	Pobre

P1: ¿Fue la pregunta de investigación y objetivos claros y apropiados? P2: ¿Estaba la población en estudio claramente especificada y definida? P3: ¿Fue la participación de al menos un 50%? P4: ¿Fueron todos los sujetos seleccionados o reclutados de la misma población o similar (incluido el mismo periodo de tiempo)? ¿Fueron los criterios de inclusión y selección especificados y aplicados? P5: ¿Hubo justificación del tamaño muestral, poder descriptivo o varianza y efectos estimados provistos? P6: Para el análisis de este paper, ¿fue la exposición de interés medida antes del *outcome*? P7: ¿Fue el marco temporal suficiente para poder esperar ver una asociación entre la exposición y el resultado de interés (en caso de existir)? P8: Para exposiciones que pueden variar en cantidad o nivel, ¿examinó el estudio diferentes niveles de exposición como los esperados en el *outcome*? P9: ¿Fue la variable independiente claramente definida, validada, fiables e implementadas constantemente entre todos los participantes? P10: ¿Fue la medida de exposición evaluada más de una vez en el tiempo? P11: ¿Fueron las medidas de resultado claramente definidas, validadas, fiables e implementadas consistentemente entre todos participantes en el estudio? P12: Fueron los evaluadores ciegos al estado de expuestos de los participantes? P13: ¿Fue la perdida de seguimiento del 20% o menos? P14: ¿Fueron los posibles confundidores medidos y ajustados estadísticamente para el impacto en la relación entre exposición y *outcome*? S: Sí; N: No; NA: No aplica; NC: No concluyente. Pobre: 0-4 criterios. Moderada: 5-10 criterios. Buena: 11-14 criterios.

**Tabla 5:** Valoración del riesgo de sesgo según la escala NIH para serie de casos

Autor/Pregunta	P1	P2	P3	P4	P5	P6	P7	P8	P9	Resultado
Van Camp *et al*. Bélgica, 2020	N	S	S	NC	N	N	NA	NA	N	Pobre
Jerjes *et al.* Reino Unido, 2010.	N	N	S	S	S	S	S	NA	S	Moderada

P1: ¿Fue la pregunta de investigación u objetivos claramente establecidos? P2: ¿Fue la población en estudio clara y totalmente descrita, incluyendo una definición de caso? P3: ¿Fueron los casos consecutivos? P4: ¿Fueron los sujetos comparables? P5: ¿Fue la intervención claramente descrita? P6: ¿Fue el *outcome* claramente definido, validado, fiable e implementado consistentemente en todo el estudio? P7: ¿Fue el periodo de seguimiento del estudio adecuado? P8: ¿Fueron los métodos estadísticos bien descritos? P9: ¿Fueron los resultados bien descritos? S: Sí; N: No; NA: No aplica; NC: No concluyente. Pobre: 0-3 criterios. Moderada: 4-6 criterios. Buena: 7-9 criterios.

Los estudios incluidos fueron realizados entre 2004 y 2022. Entre ellos, 5 casos control, 5 transversales y 2 series de casos. Solo uno de los estudios incluidos fue realizado en América (Estados Unidos), mientras que el resto se llevó a cabo en países de Europa. Los estudios tuvieron un tamaño muestral que fue desde 18 a 1146 personas. El sexo femenino fue el más afectado en todos los estudios. El promedio de edad fue de 39,5 (Rango 4-81). El subtipo de SED más prevalente en los estudios fue el SEDh. La prevalencia de desórdenes temporomandibulares varió del 26,6% al 100%. 

## DISCUSIÓN

Los pacientes con SED, especialmente aquellos con el subtipo SEDh, tienden a ser mal diagnosticados con otras patologías como fibromialgia, síndrome de fatiga crónica o depresión, debido a la gran diversidad de signos, síntomas y el impacto psicosocial que tienen en el paciente ^12^. El objetivo de esta revisión sistemática fue identificar las manifestaciones temporomandibulares en pacientes con diferentes subtipos de SED.

El colágeno y su función se ven alterados en todos los subtipos de SED, lo que puede causar síntomas en diferentes zonas del complejo orofacial. En la ATM, las estructuras fibrocartilaginosas, los ligamentos de soporte, el disco y el tejido retrodiscal están compuestos por colágeno ^9^, por lo que estos pacientes pueden manifestar patologías articulares en dicha zona.

Las manifestaciones temporomandibulares pueden ocurrir en cualquier subtipo de SED. Tres estudios de los incluidos fueron específicos para SEDh y SEDv, dos casos control y uno transversal. Bech *et al*. ^13^ concluyeron que existe una mayor prevalencia de mialgia, dolor miofascial, artralgia, dolores de cabeza atribuidos a desórdenes temporomandibulares (DTM), desplazamiento del disco y desórdenes articulares degenerativos en pacientes diagnosticados con SEDh mediante test genético. En cuanto a los pacientes con SEDv, existe una mayor prevalencia de DTM en comparación con los controles (82% vs. 24%) ^14^. Asimismo, Castori *et al*. ^15^ describen una prevalencia del 57,1% de DTM en pacientes con SEDh, según los criterios de Villefranche y Brighton.

En este estudio, los desórdenes temporomandibulares más prevalentes fueron los discales, con una marcada prevalencia de desplazamiento del disco con o sin reducción e hipermovilidad articular. En general, la hipermovilidad articular es la habilidad de una articulación para superar el rango normal de movilidad, definida como anormal cuando afecta múltiples articulaciones. Esta condición puede o no ser asintomática, y su prevalencia varía entre el 2% y el 57%, con una mayor predilección por el sexo femenino. Además, es un conocido factor de riesgo para presentar dolor musculoesquelético difuso ^(16, 17)^.

### Dolor articular/muscular

La artralgia fue reportada en 4 de los 12 estudios incluidos, con una prevalencia entre el 23,1% y el 59% entre los pacientes con SED, según los criterios RDC/TMD y DC/TMD. Por otro lado, los desórdenes musculares (miofascial) fueron reportados en cinco de los estudios incluidos para esta revisión, con una prevalencia entre el 12% y el 49%. Hagberg *et al*. ^18^ reportaron que, de 44 individuos con SED, aproximadamente la mitad presentó dolor en los músculos masticatorios. Resultados similares fueron hallados en otro estudio del mismo autor, con el 49% de los pacientes que manifestaron dolor muscular ^19^. De Coster *et al*. ^20^ reportaron un 100% de sintomatología dolorosa, del cual el 38% de los afectados padecía de dolor miofascial, y un 21% y un 51% de artralgia uni y bilateral. Resultados similares reportaron Di Giacomo *et al*. ^21^, con un 93% de pacientes que manifestaron dolor miofascial y un 70%, artralgia (uni o bilateral). Estos hallazgos pueden indicar una fuerte tendencia para desarrollar DTM en pacientes con SED.

### Hallazgos radiográficos

Tres de los estudios incluidos abordaron signos de deterioro articular mediante imagenología: radiografía ortopantomografía, tomografía de haz cónico (*cone*-*beam*) y resonancia magnética. Algunos signos descritos en imágenes 2D son remodelación prematura de la superficie temporomandibular en el 43% de los pacientes, versus el 4,3% de los controles en pacientes con SEDv ^14^. Bech *et al*. ^13^ reportaron en sus resultados que los pacientes con SEDh padecen de esclerosis subcortical en mayor proporción que los controles mediante *cone*-*beam*. En cuanto a la resonancia magnética en una serie de caso, esta fue utilizada para diagnosticar el desplazamiento del disco articular, donde hubo mayor prevalencia del estadio III (desplazamiento anterior con deformación anatómica significativa), según la clasificación de Wilkes ^22^.

## CONCLUSIONES

Las patologías temporomandibulares son habituales en pacientes con SED, especialmente en aquellos con SEDh. Las investigaciones futuras deben ser específicas para subtipos del SED, para determinar diferencias entre ellos. Además, se deben realizar estudios que evalúen la progresión de estas patologías temporomandibulares en el curso de la enfermedad, a fin de lograr un mejor entendimiento de su pronóstico en pacientes con SED. 

## Fuente de financiamiento:

Este proyecto no cuenta con fuentes de financiamiento.
